# Potential consequences of phototoxicity on cell function during live imaging of intestinal organoids

**DOI:** 10.1371/journal.pone.0313213

**Published:** 2024-11-15

**Authors:** Yuki Yokoi, Ryu Nakamura, Shuya Ohira, Shota Takemi, Tokiyoshi Ayabe, Kiminori Nakamura

**Affiliations:** 1 Innate Immunity Laboratory, Faculty of Advanced Life Science, Hokkaido University, Sapporo, Hokkaido, Japan; 2 Innate Immunity Laboratory, Graduate School of Life Science, Hokkaido University, Sapporo, Hokkaido, Japan; 3 System Development Section, Technology Solution Sector, Healthcare Business Unit, Nikon Corporation, Yokohama-City, Kanagawa, Japan; 4 Creative Research Institution, Hokkaido University, Sapporo, Hokkaido, Japan; Eötvös Loránd Research Network Biological Research Centre, HUNGARY

## Abstract

Live imaging visualizes the structure, dynamics, and function of cells and tissues to reveal the molecular mechanisms, and has contributed to the advancement of life science. In live imaging, it has been well known that there is a trade-off between higher-resolution analysis and cell damage caused by light illumination, i.e., phototoxicity. However, despite the risk of unknowingly distorting experimental results, phototoxicity is an unresolved issue in live imaging because overall consequences occurring inside cells due to phototoxicity remains unknown. Here, we determined the molecular process of phototoxicity-induced cell damage systematically under low- and high-dose light illumination conditions by analyzing differential gene expression using RNA-sequencing in a three-dimensional organoid of small intestinal epithelial cells, enteroid. The low-dose light illumination already induced various abnormalities in functional molecules involved in the response to reactive oxygen species generated by the excitation of fluorescent dyes, intracellular metabolism, mitosis, immune responses, etc., at mRNA expression level. Together with the behavior toward apoptosis caused by high-dose light illumination, the light dose-dependent progression of intracellular damage was revealed. About visible impairment of intestinal epithelial function, failures in both the structure-forming ability of enteroids and Paneth cell granule secretion were observed under high-dose light illumination, while the drug efflux was not disturbed despite abnormal drug efflux transporter mRNA expression. Based on the gene expression profiles, we comprehensively clarified phenomena in the cells at mRNA level that cannot be recognized both morphologically and functionally during live imaging, further providing a new insight into the risk of phototoxicity. This study warns from the aspect of mRNA expression that awareness of phototoxic artifacts is needed when analyzing cellular function and the mechanism in live imaging.

## Introduction

Live imaging is becoming increasingly important in a wide range of research fields in life sciences including medicine, as it allows us to observe cells and biological tissues while they are alive to reveal cell morphology, function, and the molecular mechanism. In biological studies using microscopes, long exposure time and high light intensity are often required to obtain high-resolution images and observe deep tissues. Phototoxicity, which disrupts physiological behavior of cells due to reactive oxygen species (ROS) generated when light illumination excites fluorescent molecules during live imaging, has become a major problem [1–4]. The effects of phototoxicity are not immediately visible, creating artifacts in experimental results and unwittingly leading to erroneous conclusions. In addition, phototoxicity is known to depend on various factors such as cell types, experimental systems, and wavelength and doses of excitation light, and there has been no standardized criterion for assessment of phototoxicity. Thus, phototoxicity is a common concern among researchers conducting live imaging in a wide range of life science fields [5]. Phototoxicity has been analyzed and evaluated based on morphological signs such as membrane blebbing and vacuole formation, as well as mitotic delay and arrest, abnormal cell motility, cell death [1, 4, 6–8], and also behavioral changes in the organism [9]. However, because the whole picture of the phenomena that occur inside cells due to phototoxicity is not fully known, distinguishing the physiological dynamics of cells from the behavior caused by phototoxicity can be difficult, leading to incorrect experimental results. Phototoxicity is an unsolved important challenge in live imaging.

To reveal the dynamics and functions of various cell types by live imaging, organoids, which are three-dimensional structures that maintain the morphology and function similar to organs or tissues *in vivo* and are formed from stem cells and progenitor cells through a self-organization process, have been widely used [10–13]. The small intestinal epithelial organoids, enteroids, consisting of all intestinal epithelial cell lineages, including terminally differentiated cells with absorptive enterocytes, goblet cells, enteroendocrine cells, and Paneth cells, and intestinal epithelial stem cells (ISCs) providing all these cells have been used to analyze functions of intestinal epithelial cells [14, 15]. Confocal microscopy is particularly useful and widely used for observing thick samples such as organoids, tissues, and organisms. While enteroids, a three-dimensional structure composed of heterogeneous epithelial cell populations, mimic the structure and function of physiological small intestinal epithelium, it has a greater thickness than the cultured cell lines usually used in live imaging, thus requiring long exposure to strong light to reach deep into the epithelium and to observe cell dynamics in a spatially and temporally. Therefore, a further understanding of the effects of phototoxicity is required to reveal cell functions and their mechanisms using enteroids. This study aimed to systematically clarify the alteration of cell function during the process of phototoxicity from early to late stages in live imaging of enteroids using a confocal microscope by comprehensively evaluating mRNA expression, which is most sensitive to environmental changes in cells induced by external stimulation.

By RNA-sequencing analysis (RNA-seq) of enteroids with fluorescent imaging under two different light exposure conditions: intermittent-scan with low light dose and continuous-scan with high light dose, we found that mRNA expression involved in ROS scavenging, metabolism, membrane transport, mitochondria, cell division, and immune response changes with low light dose already compared to non-illumination control, and that mRNA expression involved in cell death is affected with high light dose. Furthermore, analyses of the phototoxic effects on epithelial cell function revealed that the structure-forming ability of small intestinal epithelial cells and the granule secretion from Paneth cells, which is an innate immune response, were not impaired by intermittent-scan, whereas impaired by continuous-scan and the drug efflux ability was less affected by both scans. Here we show a scientific basis for understanding what actually happen and what can happen inside cells during live imaging, by clarifying transitions in gene expression from early stages of phototoxicity toward cell death.

## Materials and methods

### Mice

Eight-week-old male C57BL/6JJcl mice were purchased from CLEA Japan. For collecting small intestinal tissue, mice were euthanized by cervical dislocation, and all efforts were made to minimize suffering. All animal experiments were approved by the National University Corporation Hokkaido University Animal Experimentation Committee (Approved No. 23–0057). All experiments were performed in accordance with relevant guidelines and regulations of Hokkaido University. This study was also carried out in compliance with the ARRIVE guidelines.

### Enteroid preparation

Small intestinal crypt isolation and enteroid culture were performed as described previously [16]. Briefly, mouse proximal small intestine was incubated in 30 mM EDTA with Ca^2+^- and Mg^2+^-free Hank’s balanced salt solution (HBSS) for 10 min at room temperature. The tissue was shaken vigorously in fresh HBSS to release crypts. Isolated crypts were embedded in Matrigel (Corning) and enteroid culture medium containing growth factors (EGF, R-Spondin 1, Noggin) was added. Culture medium was changed every other day. For phototoxicity analysis of enteroid fluorescent imaging, Paneth cell granules and plasma membrane were stained with Zinpyr-1 (Santa Cruz Biotechnology) and CellMask^TM^ Deep Red Plasma Membrane Stain (CellMask, Thermo Fisher Scientific), respectively. Enteroids were incubated in 10 μM Zinpyr-1 for 16 h at 37°C, 5% CO_2_. After Zinpyr-1 staining, enteroids were collected by depolymerization of the Matrigel with ice-cold Cell Recovery Solution (Corning) and resuspended with pre-warmed culture medium with 25 μg/mL CellMask and incubated for 10 min at 37°C, 5% CO_2_. Stained enteroids were replaced onto glass-based dish with fresh Matrigel.

### Enteroid imaging and RNA-sequencing analysis

For RNA expression analysis of light-illuminated enteroids, enteroids at day 3 or 4 of culture stained by Zinpyr-1 / CellMask were transferred onto Culture-Insert 2 Well in μ-Dish 35 mm (Ibidi) at 300 enteroids/well. The dish was set on the Stage Top Incubator (Tokai Hit) which was thermally-controlled enclosure set at 37°C, 5% CO_2_. To illuminate light to all enteroids on the dish, a 3 mm × 6 mm area were scanned with 488 and 647 nm laser by using Resonant (Intermittent) or Galvano (Continuous) scanner of a confocal microscope (A1, Nikon) equipped with 0.75 NA objective lens (Plan Apo 20X DIC M N2, Nikon). The scan settings and light dose were described Table 1.

**Table 1:** Imaging parameters.

Parameter	Low-dose light illumination	High-dose light illumination
Light source	Diode laser	
Wavelength	488 nm / 647 nm	
Scan mode	Intermittent	Continuous
Pixel dwell	0.26 μsec	3.8 μsec
Scan number	600	600
Light dose	71 J/cm^2^	1063 J/cm^2^

Light-illuminated enteroids were harvested using Cell Recovery Solution. Total-RNA was extracted from light-illuminated enteroids for three independent imaging experiments using RNeasy Mini kit (Qiagen) and quality checked using a BioAnalyzer (Agilent). Polyadenylated RNAs were enriched using NEBNext^®^ Poly(A) mRNA Magnetic Isolation Module (New England BioLabs). cDNA libraries were prepared using NEBNext® UltraTMII Directional RNA Library Prep Kit according to manufacturer’s protocols (New England BioLabs). The libraries were sequenced using a NovaSeq 6000 instrument (Illumina) with 150 bp paired-end reads. The read quality was assessed using the FastQC ver.0.11.7. The raw sequence data were trimmed for quality using Trimmomatic ver.0.38 and trimmed data were mapped to the mouse reference genome by HISAT2 ver.2.1.0. The read counts were calculated using featureCounts ver.1.6.3 and were normalized to transcripts per million (TPM) by EdgeR ver.3.36.0. The TPM values were scaled to log_2_(TPM+0.01) for downstream analysis. For k-means clustering analysis, the TPM values were standardized to Z-scores, and the transcript expression patterns were estimated by k-means clustering in R package. Functional annotations of gene ontology (GO) processes were determined and GO enrichment analysis were conducted by Metascape [17].

### Evaluation of enteroid formation ability, drug efflux, and Paneth cell granule section

To evaluate the effect of light-illumination to epithelial function of enteroids, fluorescent labeled isolated crypts and enteroids were exposed to excitation light for 10 min under conditions shown in Table 1 using confocal microscope (Nikon, A1) with 0.95 NA objective lens (CFI Apo LWD 20X WI λS, Nikon). Along with the fluorescence images, differential interference contrast (DIC) images were obtained. The isolated crypts stained with Zinpyr-1 and CellMask were cultured for 5 days after light-illumination on Cell imaging dish (Eppendorf). The number of enteroids and protrusions were counted every day to assess the stem cell activity. Drug efflux of enteroids were assessed as previously described [18]. Briefly, enteroids which were single-stained with CellMask and seeded on the collagen-coated 8 well chambered coverglass (Matsunami) were illuminated to laser light for 10 min under conditions shown in Table 1. The light-illuminated enteroids were treated with 1 μM Rhodamine 123 (Rh123, Sigma-Aldrich) at 37°C, 5% CO_2_ for 90 min. The images of the enteroids before and 90 min after adding Rh123 were obtained. Rh123 intensities of enteroid lumen were measured using NIS-Elements AR ver.5.11 (Nikon). To evaluate Paneth cell granule secretion, enteroids stained with Zinpyr-1 and CellMask were stimulated with 10 μM carbamylcholine (CCh, Sigma-Aldrich) and time-lapse imaging were performed under conditions shown in Table 1 for 10 min at 37°C, 5% CO_2_. For quantifying Paneth cell granule secretion, Paneth cell granule area in the DIC images was measured before and 10 min after CCh stimulation by using an image analysis software, NIS-Elements AR (Nikon). The percent decrease area of Paneth cell granules before and after stimulation was calculated as Paneth cell granule secretion (%) described previously [16].

### Statistical analysis

All statistical computations were performed using GraphPad Prism 9 (GraphPad Software). Data comparing three groups were analyzed by one- or two-way ANOVA followed by Tukey’s multiple comparisons test. Differences between groups were considered significant if P-values were < 0.05.

## Results

### Phototoxicity-induced abnormalities in gene expression arise by low light exposure

**Fig 1:**
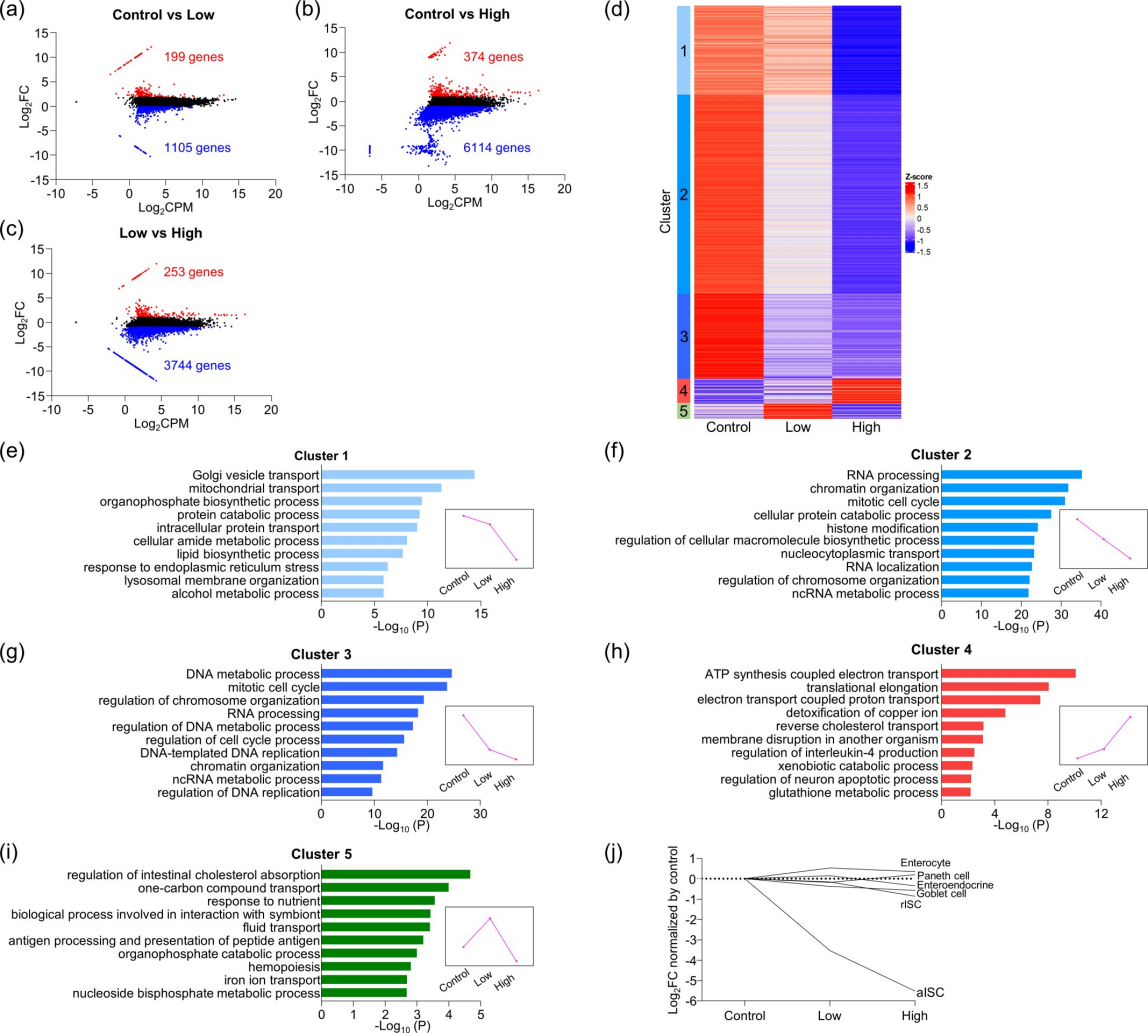
Transcriptome profiling of light-illuminated enteroids. (a–c) MA-plot showing DEGs in light-illuminated enteroids between control, low-dose light illumination, and high-dose light illumination. (d) Gene expression profiling among each light illumination condition by k-means clustering analysis (k = 5, 1000 repetitions). Heatmap represents up- and downregulated genes within five clusters for individual experimental samples. (e)–(i) GO enrichment analysis result of genes in each cluster. The GO terms from biological process within the top 10 accumulative hypergeometric p-values were represented. All GO terms significantly enriched (p < 0.01) were listed in S1 Table. Magenta line graphs represent variation patterns of mean gene expression under light illumination in each cluster. (j) mRNA expression of major cell lineage markers (aISC: *Olfm4*, rISC: *mTert*, enterocyte: *Alpi*, enteroendocrine: *Sct*, goblet cell: *Muc2*, Paneth cell: *Defa3*) of light-illuminated enteroids.

To comprehensively and systematically identify the phenomena that occur in cells by light illumination in fluorescent live imaging, mRNA expression of the enteroids exposed to low-dose (intermittent-scan; 71 J/cm^2^) and high-dose (continuous-scan; 1063 J/cm^2^) light (S1 Fig) and non-illumination control (control) were analyzed by RNA-seq analysis. We detected 11,496 genes with transcript per million (TPM) > 5 in at least one of the three groups, and genes that satisfied log_2_|Fold change| > 1 were defined as differentially expressed genes (DEGs). The total number of DEGs due to light illumination was 6,786 genes (59.0%). Low-dose light illumination increased the expression of 199 genes and decreased 1,105 genes compared to control (Fig 1A), while high-dose light illumination increased 374 genes and decreased 6,114 genes (Fig 1B). In addition, the expression of 253 genes increased and 3,744 genes decreased in high-dose light illumination compared to low-dose light illumination (Fig 1C). Accordingly, 11.3% of the total mRNA expression was affected by intermittent-scan with low-dose light, whereas 56.4% changed by continuous-scan with high-dose light.

Next, to clarify the cell functions affected by illumination with each light dose, k-means clustering was performed, and DEGs were classified into five clusters based on the pattern of gene expression across each light exposure condition (Fig 1D). When we performed GO enrichment analysis on each cluster (Fig 1E–1I), a group of genes, cluster 1, which drastically decreased by continuous-scan with high light dose compared to both control and intermittent-scan with low light dose, were extracted and annotated with organelle transport (e.g. "Golgi vesicle transport"), intracellular metabolism (e.g. "protein catabolic process"), and apoptosis (e.g. "response to endoplasmic reticulum stress") (Fig 1E). On the other hand, genes in cluster 2, the expression level decreased in a light dose-dependent manner, and genes in cluster 3, the expression level decreased particularly by low-dose light illumination, were both annotated with "RNA processing", "chromatin organization", "DNA metabolic process", and "mitotic cell cycle”, all involved in cell proliferation and nucleic acid and protein synthesis (Fig 1F and 1G). In addition, genes in cluster 4, classified as light-dependent increase were annotated with mitochondrial oxidative stress responses induced by elevated intracellular ROS, such as "ATP synthesis coupled electron transport" and "glutathione metabolic process" (Fig 1H). Genes in cluster 5 that were markedly increased only by low-dose light illumination were enriched in "regulation of intestinal cholesterol absorption", "one-carbon compound transport", etc., all related to the transport and metabolism of nutrients (Fig 1I). Furthermore, low-dose light illumination activated genes involved in the immune response, such as "biological process involved in interaction with symbiont" and "antigen processing and presentation of peptide antigen" (Fig 1I). Thus, not only continuous-scan with high light dose but also intermittent-scan with low light dose caused differential expression of many genes involved in various functions of the small intestinal epithelial cells constituting enteroids including ROS response, intra- and extracellular transport, immune response, and further most of cell function.

To determine effects of light-illumination on each cell lineage in the enteroids, we next investigated the expression of marker genes for actively proliferating ISCs (aISCs), reserve (ISCs) rISCs, goblet cells, enteroendocrine cells, and Paneth cells [19, 20]. The mRNA and protein expression of *Olfm4*, a marker of aISCs that are actively dividing, markedly decreased in a light dose-dependent manner (Figs 1J and S2). In contrast, expression levels of markers for rISCs that normally remain in the quiescent stage of cell division and all terminally differentiated cells including Paneth cells showed almost no difference with any doses of light compared to control (Fig 1J). Accordingly, rapidly dividing aISCs were more sensitive to phototoxicity even with lower dose light than the other epithelial cells, which are in a quiescent state, and the stem cell activity was impaired first.

### High-dose light illumination severely impairs stem cell activity to disrupt the structure formation ability of intestinal epithelial cells

**Fig 2:**
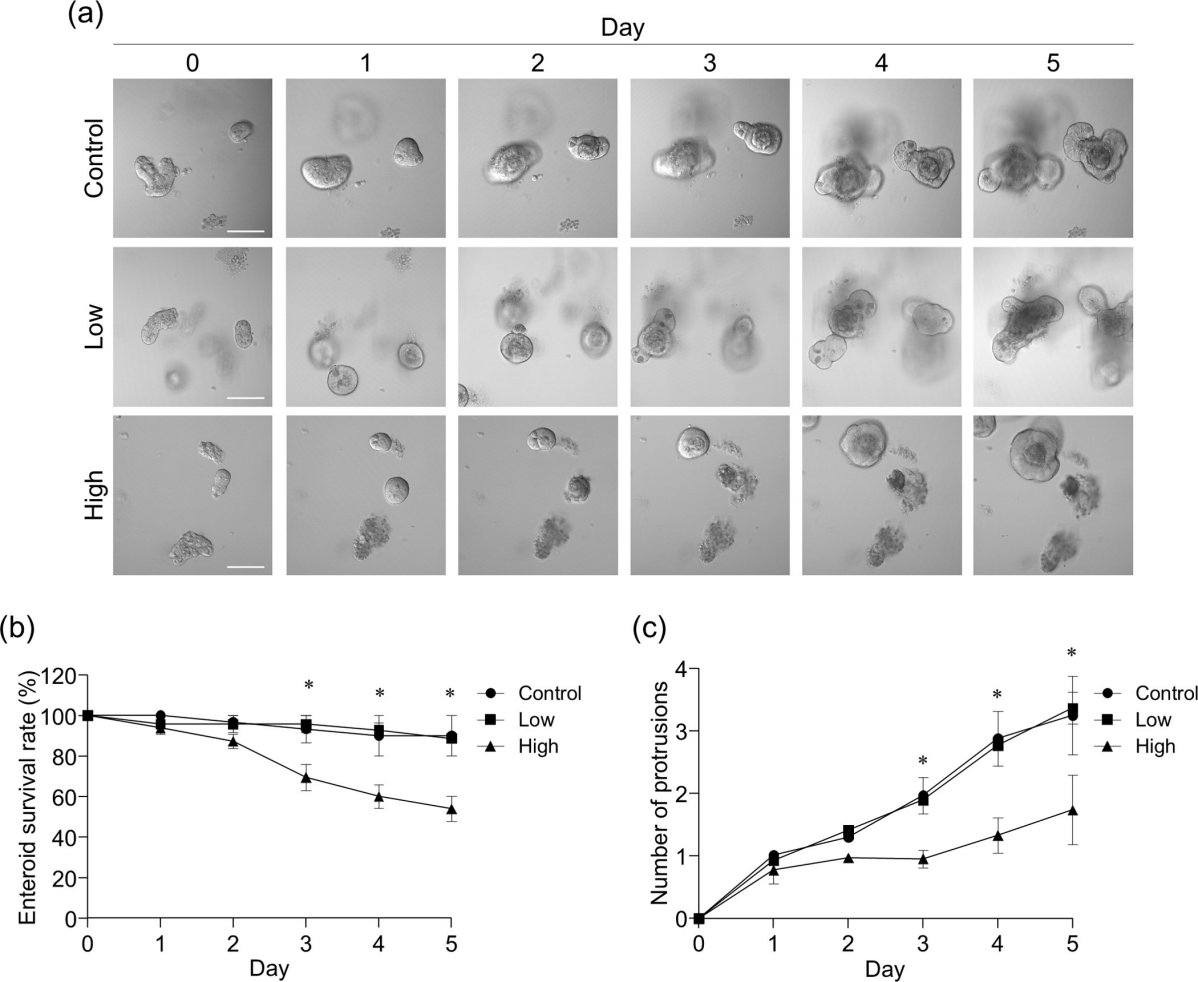
Disruption of enteroid formation ability due to light illumination. (a) Representative DIC images of the culture progress of the light-illuminated enteroids by low- or high-dose light illumination. Scale bars: 100 μm. (b) Daily progression of enteroid survival rate (%) after light illumination at day 0. (c) Protrusion number of enteroids exposed to light on day 0. The values were depicted as mean ± standard error of the mean for three independent experiments. * represents significant difference for high-dose light illumination values as compared to both control and low light dose condition (P < 0.05).

Since the expression level of the aISC marker *Olfm4* was markedly decreased by light illumination, enteroid formation ability, which reflects the self-renewal and differentiation of aISCs among the small intestinal epithelial cell functions, was first evaluated. When isolated crypts were exposed to light with intermittent-scan or continuous-scan on the first day of enteroid culture and followed the subsequent culture progress, the number of enteroids having budded crypt-like structure (protrusions) decreased significantly after day 3 of culture by high-dose light illumination compared to control and low-dose light illumination (Fig 2A and 2B). In addition, although the number of protrusions counted as an index of stem cell activity showed no difference between control and low-dose light illumination, enteroids exposed to high-dose light had significantly decreased number of protrusions after day 3 compared to control and low-dose light (Fig 2A and 2C). These results indicated that low-dose light illumination decreases Olfm4 expression without the effect of enteroid formation, while high-dose light illumination impairs not only Olfm4 expression but also the self-renewal and differentiation of aISCs. Moreover a reduction of fluorescent intensity, i.e., bleaching of fluorescent dyes, which is widely known as an indicator of excessive light illumination, was not observed in intermittent-scan isolated crypts, whereas the fluorescent intensity was significantly reduced in continuous-scan (S3 Fig).

### Visible morphological disruption in intestinal epithelial cell function occurs with high-dose light illumination

**Fig 3:**
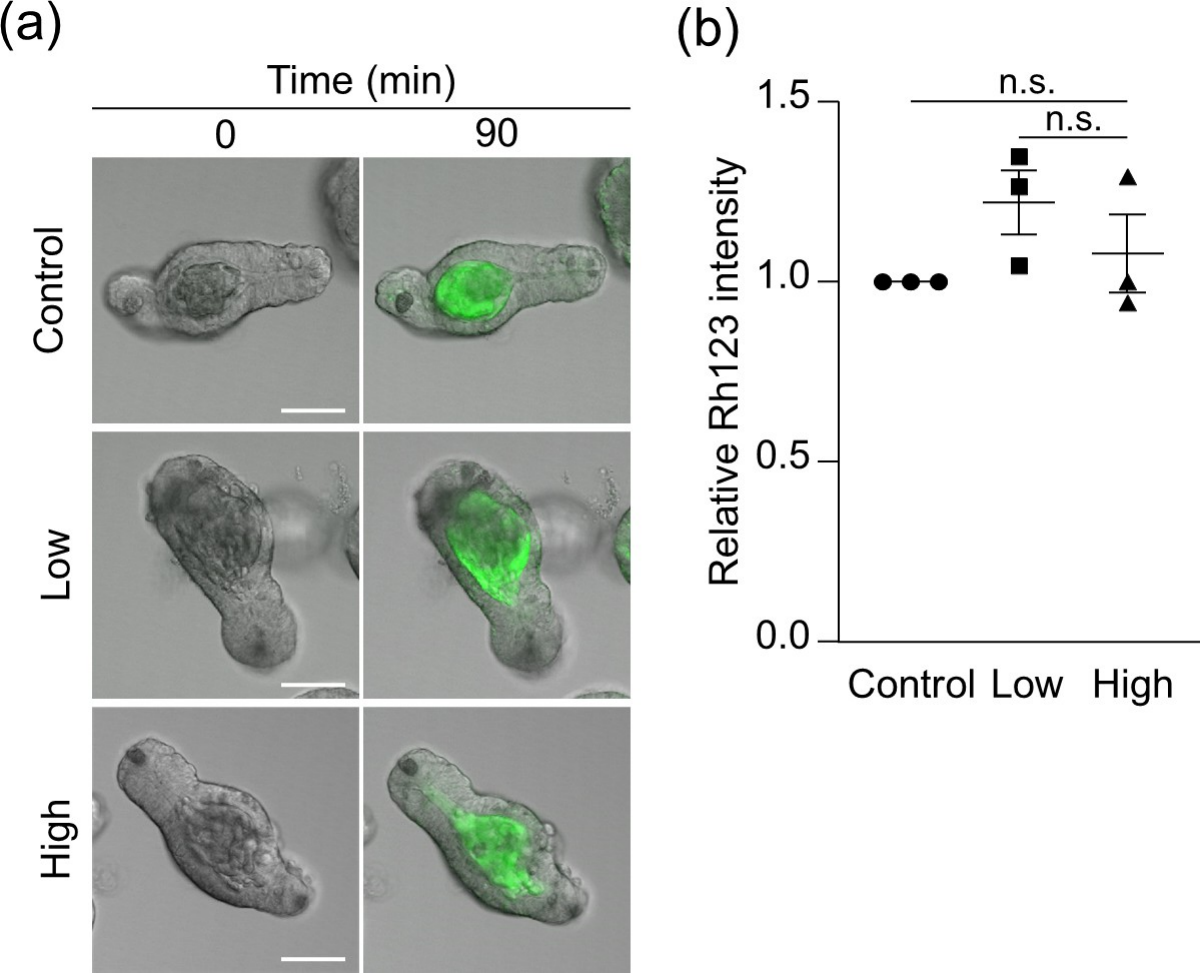
Drug efflux in light-illuminated enteroids. (a) Representative merged fluorescent and DIC images of the light-illuminated enteroids by low- or high-dose light illumination 90 min after adding Rh123 (green), compared with control enteroids. Scale bars: 50 μm. (b) The relative Rh123 intensity is fluorescent intensity at the lumen of light-illuminated enteroids 90 min after adding Rh123 normalized by control. The values were depicted as mean ± standard error of the mean for three independent experiments. n.s., not significant.

Finally, we investigated the effects of light illumination on the function of terminally differentiated cells in enteroids. Small intestinal epithelial cells are known to efflux drugs to the outside of the body via the drug efflux transporter P-glycoprotein (P-gp) [21]. When Rh123, a fluorescent substrate of P-gp, was added to the enteroid culture medium to evaluate the effects of light illumination on the drug efflux into the intestinal lumen, the Rh123 intensity in the enteroid lumen of low- and high-dose light illumination after 90 min was equivalent to that of control (Fig 3A and 3B). These results indicated that the drug efflux of the small intestinal epithelial cells was maintained even with high-light illumination.

**Fig 4:**
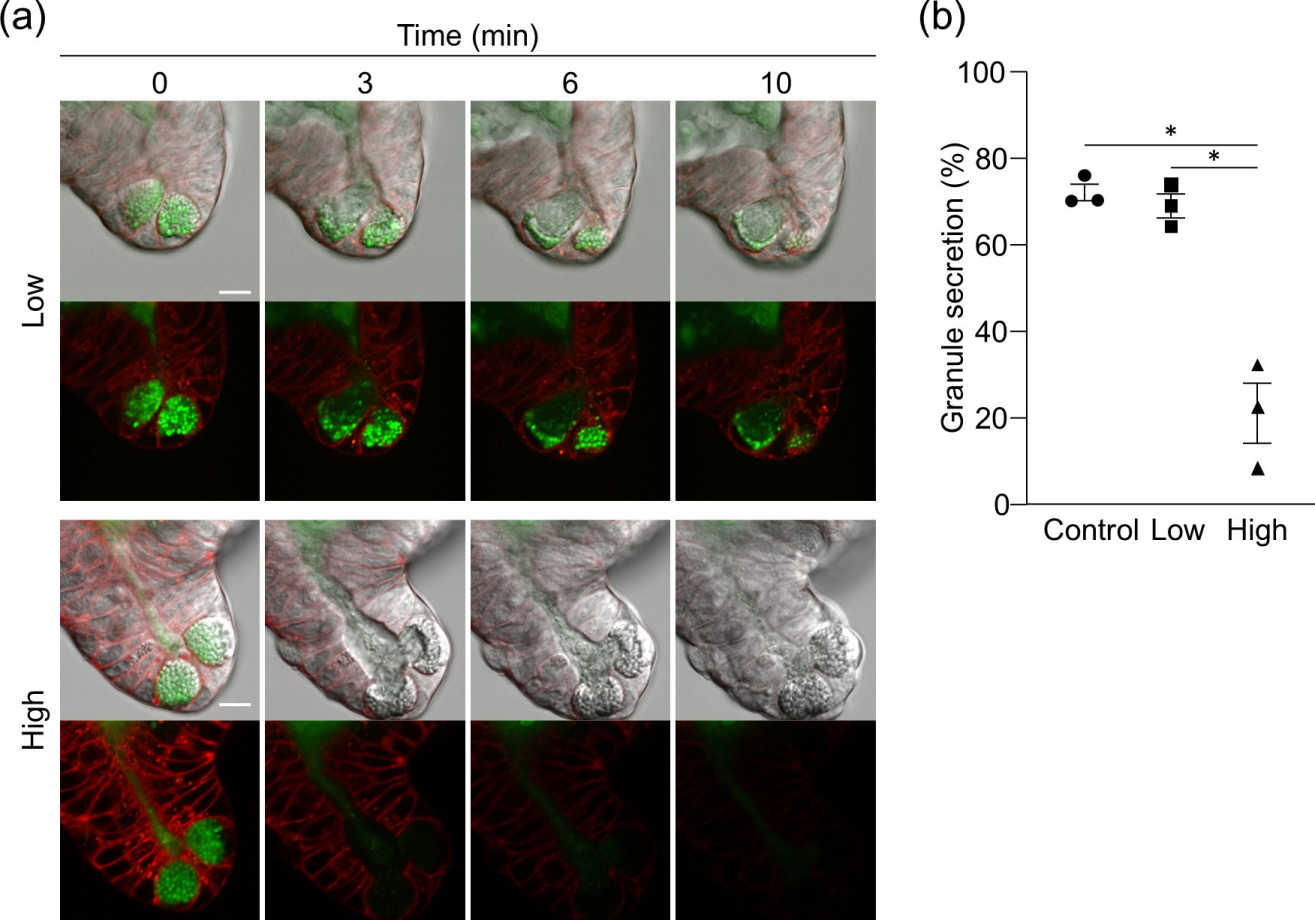
Inhibition of Paneth cell granule secretion by light illumination. (a) The representative fluorescent and DIC images of Paneth cells acquired by low or high-dose light illumination for 10 min after adding 10 μM CCh. Paneth cell granules and cell membrane were stained by Zinpyr-1 (green) and CellMask (red), respectively. Scale bars: 10 μm. (b) Percent granule secretion 10 min after adding CCh. The values were depicted as mean ± standard error of the mean for three independent experiments. *P < 0.05.

Paneth cells are secretory cells having cytoplasmic granules approximately 1 μm in diameter located at the base of small intestinal crypts, and contribute to innate enteric immunity by secreting various microbicidal substances such as antimicrobial peptides, mostly α-defensins, into the intestinal lumen [22, 23]. When a cholinergic ligand, CCh which is known to induce Paneth cell granule secretion, was added to enteroid culture medium to evaluate the effect of light illumination on the secretion, Paneth cells in control and low-dose light illuminated enteroids vigorously secreted granules for 10 minutes. In contrast, Paneth cell granule secretion in high-dose light illumination was suppressed after about 3 min from adding CCh, and completely stopped at 6 min (Fig 4A and S1 Video). The percent granule secretion of Paneth cells in control and low-dose light illumination enteroids was the same level, while that in high-dose light illumination was significantly lower than the other two groups (Fig 4B). These results indicated that, in the conditions of this study, morphologically visible impairment in small intestinal epithelial cell function was not observed under low-dose light illumination, whereas was more pronounced under high-dose light illumination.

## Discussion

**Fig 5:**
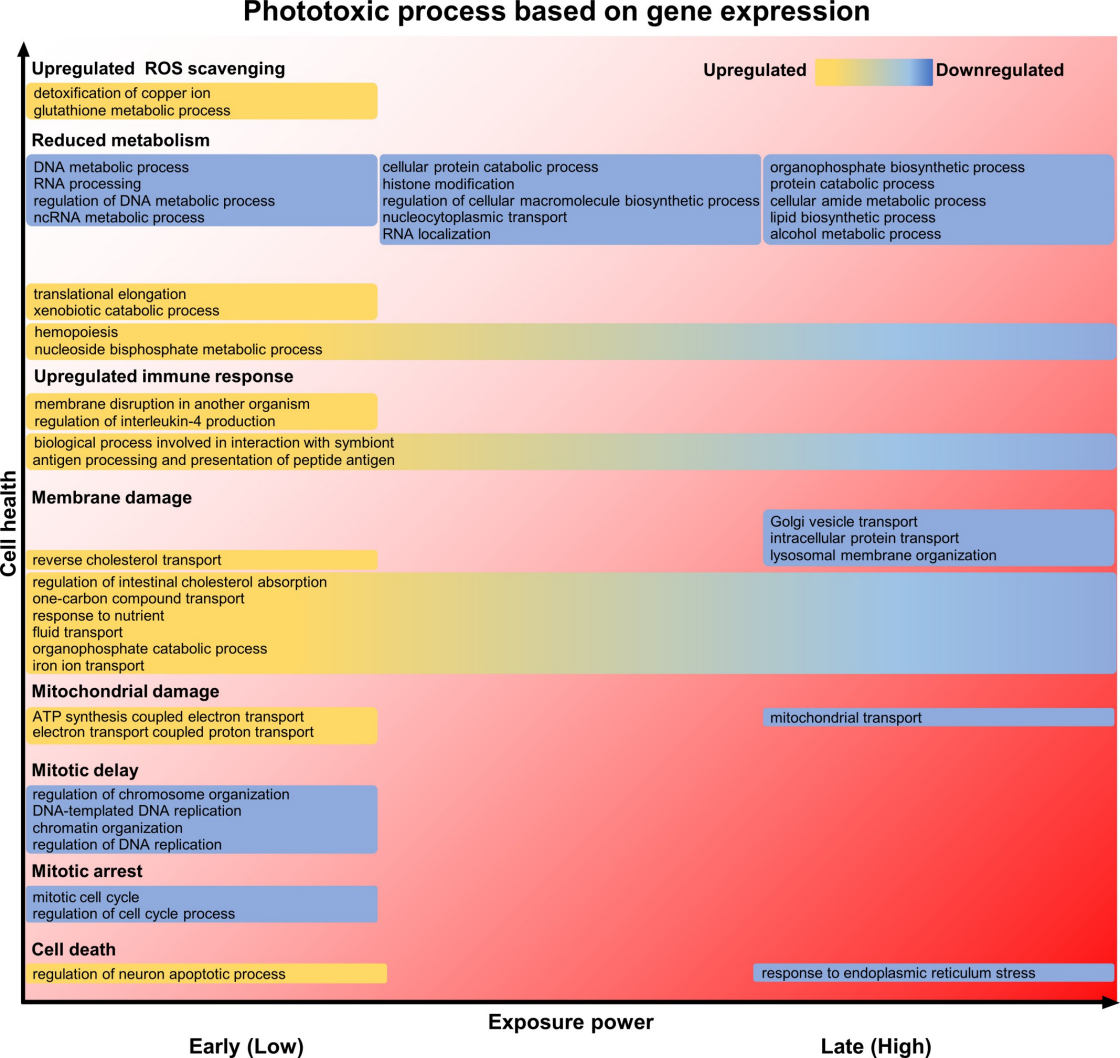
Process of phototoxicity based on gene function in small intestinal epithelial cells. From the GO enrichment analysis, the phototoxicity process was estimated from the biological process associated with the extracted pattern of genetic variation due to two levels of light illumination, referring to the phototoxicity process proposed by Laissue *et al*. [5].

Live imaging, which visualizes dynamics of cells, tissues, and individual organisms in real time, is an indispensable method for elucidating structure, function, and the molecular mechanisms of cells, and has been greatly advancing the field of life science [24]. On the other hand, it is widely recognized by researchers conducting live imaging that phototoxicity, toxic effects of light on cells, can impair physiological behavior of cells so that the doses of light illumination to cells should be reduced to minimize phototoxicity. There is a trade-off between phototoxicity and high-resolution images, which often makes live imaging difficult. However, the whole picture of what kind of specific molecular changes occur step-by-step inside cells when exposed to light and how they affect cell functions in live imaging is not fully understood. In this study, we attempted to understand the process of phototoxicity-induced cell damage at the molecular level by comprehensively comparing mRNA expression of enteroids under two light illumination conditions, low light dose by intermittent-scan and high light dose by continuous-scan in live imaging. It has been known that phototoxicity is caused by ROS generated with excitation of fluorescent molecules, leading to cell death through enhancement of ROS scavenging, reduction of intracellular metabolism, plasma membrane damage due to spontaneous increase of intracellular Ca^2+^, mitochondrial damage due to membrane depolarization, and mitosis delay or arrest [25–30]. Based on our results extracted by GO annotation analysis with enteroid genetic variation pattern, we attempted to systematize consequences of the intracellular molecular process of phototoxicity comparing the gold standard for phototoxicity cascade previously reported by Laissue et al. [5], and summarized in Fig 5. Low-dose light exposure activated the biological process involved in ROS scavenging, including *Metallothionein*, known to be involved in ROS inactivation [31, 32], and *Gclc* and *Gstm1*, involved in glutathione synthesis (Figs 1H and 5 and S1 Table: cluster 4). In addition, because DEGs associated with various biological processes responsible for intracellular metabolism, various transporters and receptors expressed on the plasma membrane, and mitochondrial function was already fluctuated by low-dose light exposure (Figs 1F–1I and 5 and S1 Table; cluster 2–5), it is suggested that even low-dose light affects cell function related to almost all stages of the ROS-induced phototoxic cascade proposed previously. Moreover, "membrane disruption in another organism" and "regulation of interleukin-4 production", which are gene functions related to immune response, were up-regulated in low-dose light exposure, i.e., relatively early stage of phototoxicity (Figs 1H, 1I and 5 and S1 Table; cluster 4, 5), indicating that ROS produced by light illumination may activates epithelial immune response [33]. On the other hand, the expression of genes involved in biological processes such as "response to endoplasmic reticulum stress" including genes related to apoptosis was specifically down-regulated by high-dose light exposure (Figs 1E and 5 and S1 Table; cluster 1). In particular, the expression levels of *Ripk1*, which is known to inhibit necrosis and apoptosis [34], decreased in a light dose-dependent manner (S2 Table; cluster 2), suggesting that phototoxicity cascade, which has already initiated with low light dose, is irreversible toward cell death with high light dose. Although this study revealed gene functions involved in the mRNA expression disrupted by phototoxicity finally leading to cell death, the details of the time sequence of the mRNA expression and the interactions between the molecules remain to be determined.

By using enteroids, this study simultaneously assessed phototoxicity against actively proliferating stem cells and terminally differentiated cells of multiple lineages that are arrested in mitosis. It has been known that mitotic cells are particularly sensitive to phototoxicity [35], consistent with our results that the expression of the aISCs marker *Olfm4* is already markedly down-regulated with low-dose light illumination compared to other cell lineage markers. On the other hand, the high-dose light illumination caused prominent effects on the function of certain terminally differentiated cells. The evaluation system for drug efflux of the intestinal epithelial cells using enteroids was established [36, 37], and functional analysis of epithelial drug transport dynamics has been conducted by imaging organoids derived from various organs and tissues such as the liver and blood-brain barrier [38]. In this study, the drug efflux transporter P-gp expressed in small intestinal epithelial cells, was not impaired by both low- or high-dose light illumination, while the expression level of *Abcb1a*, which encodes P-gp, was decreased in a light dose-dependent manner (S2 Table; Cluster 3), indicating that excessive laser exposure may unwittingly distort the results of molecular mechanism analysis of in live imaging. Paneth cells secrete granules rich in various microbicidal substances, such as the antimicrobial peptide α-defensin, into the intestinal lumen, and are responsible for innate enteric immunity and regulation of the intestinal microbiota composition [39–42]. α-Defensin abnormalities have been reported to be involved in a variety of diseases such as inflammatory bowel disease and depression [43–48]. We have previously established the evaluation system by visualization and quantification of Paneth cell granule secretion using enteroids, and clarified Paneth cell secretory dynamics to contribute to understanding mechanisms in intestinal immune system [16, 49–51]. Among the genes that were remarkably down-regulated only by continuous-scan, genes involved in vesicle transport leading to exocytosis, such as *Vamp3*, a SNARE protein, and *Rab1a*, a small G protein [52, 53] both associated with "Golgi vesicle transport" were found (S1 Table; cluster 1), consistent with suppression of Paneth cell granule secretion only under continuous-scan with high light dose. Many of the disturbances in gene expression associated with small intestinal epithelial cell function occurred in intermittent-scan with low light dose clearly indicates the need for careful condition settings such as laser power and exposure time according to the purpose of the experiment.

Organoids, including enteroids, mimic the three-dimensional structure of organs and tissues, and advances in three-dimensional imaging using confocal microscopy and multiphoton microscopy have allowed us to observe complex tissue structures and cell-cell interactions [12]. In this study, ISCs were dramatically injured with low-dose light illumination, indicating that live imaging of the organoids, whose basis is morphogenesis by tissue stem cells or induced pluripotent stem cells, light illumination conditions should be carefully set to minimize photodamage of the stem cells. Recently, *in vivo* imaging with multiphoton excitation microscopy has been performed to elucidate the molecular dynamics of intestinal tissues and cells by direct observation [54–58]. Furthermore, imaging techniques using visible to far-red light, such as optical CT and optical topography, which noninvasively obtain cross-sectional images of the body for diagnostic purposes, have become indispensable for basic life science research and medicine. The phototoxicity of these techniques has not yet been fully investigated, and this remains a challenge for the future.

This study set two different doses of light, low (intermittent) and high (continuous), within the range that is actually used in fluorescent live imaging with a three-dimensional structure of cells aiming to obtain high-definition images. There are two major methods for modulating the light dose illuminated to cells: adjusting the laser power or adjusting the pixel dwell time under fixed laser power. In this study, we set low- and high-dose light illumination conditions by adjusting the pixel dwell time, which is usually used to reduce phototoxicity. On the other hand, it has been reported that even if the same dose of light is illuminated to cells using two different illumination methods, intermittent-scan or continuous-scan, by adjusting pixel dwell time, phototoxicity is reduced in intermittent-scan with a shorter pixel dwell time [28]. Therefore, the mRNA expression alterations identified in this study may also be influenced by the difference in illumination methods. Although it is well known that longer wavelength of excitation light causes less damage to cells [1], a multiple staining by simultaneous excitation with 488 nm and 647 nm laser light with better fluorescence separation is often used to analyze interactions between organelles or molecules and adopted in this study.

Based on the findings of this study that light illumination, even at low light dose, alters mRNA expression, it is useful to consider the following actions to reduce phototoxicity and to perform proper live cell imaging. Those include using wide-field imaging rather than point scanning with a laser, careful selection of bright fluorophores requiring lower light dose, use of high-sensitive detectors, widening the confocal aperture to capture more light, and use of digital enhancement methods or AI to improve image quality acquired at low light dose. From a different perspective, it may be useful to see how low dose of light phototoxicity begins, what the maximum amount of light illumination that does not damage the major function of each cell is, and how the wavelength- and pixel dwell time affect cells for understanding phototoxicity itself more deeply or developing a novel microscope system that enables to reduce phototoxicity. The various alterations in mRNA expression by low-dose light illumination, as revealed in this study, will greatly contribute to the recognition based on a systematic understanding of the risks of phototoxicity in all areas where light illumination is applied to biological tissues and cells, and provide a new benchmark for minimizing artifacts in live imaging in broad life science fields for obtaining true new biological knowledge.

## Supporting information

S1 Fig Enteroids imaged by two light illumination conditions. Representative images of enteroids acquired by low-dose light (intermittent-scan) and high-dose light (continuous-scan) illumination conditions. Enteroids were stained by Zinpyr-1 (Paneth cell granule, green) and CellMask (cell membrane, red). Scale bars: 10 μm.
(TIF)

S2 Fig Cell lineage marker expression in light-illuminated enteroids. (a) Representative immunofluorescent staining images of Olfm4 (aISC, red) and α-defensin (Paneth cell, green) with DAPI (nucleus, blue) counterstaining in enteroids with each light-illuminated condition. Scale bars: 50 μm. (b) The Olfm4 intensities of light-illuminated enteroids. The values were depicted as mean ± standard error of the mean for three independent experiments. *P < 0.05.
(TIF)

S3 Fig Fluorescence bleaching by light illumination of isolated crypts. (a) Representative time-lapse images of isolated crypts stained with CellMask exposed to intermittent- or continuous-scan. Scale bars: 50 μm. (b) The time course of CellMask intensity changes in light exposed isolated crypts relative to time = 0 min. The values were depicted as mean ± standard error of the mean.
(TIF)

S1 Video Time-lapse images of Paneth cell granule secretion induced by 10 μM CCh acquired by intermittent- or continuous-scan. CCh was added to the culture medium at 10 sec. Paneth cell granules and cell membrane were stained by Zinpyr-1 (green) and CellMask (red), respectively. Fluorescent and DIC images were overlayed. Real acquisition time are represented by h:mm:ss (hour:minute:second). Scale bar: 10 μm.
(MP4)

S1 Table All GO terms significantly enriched in each cluster. GO terms significantly (P < 0.05) extracted by GO enrichment analysis are listed. Each cluster is divided into tabs.
(XLSX)

S2 Table Genes of each cluster classified by k-means clustering analysis. Gene groups classified by k-means clustering are listed in tabs. TPM values for each gene were standardized to Z-score.
(XLSX)
