# Supplementary figures and images for: Potential consequences of phototoxicity on cell function during live imaging of intestinal organoids
Source: PLoS One. 2024 Nov 15;19(11):e0313213. doi: 10.1371/journal.pone.0313213 (PMC11567556; doi:10.1371/journal.pone.0313213)

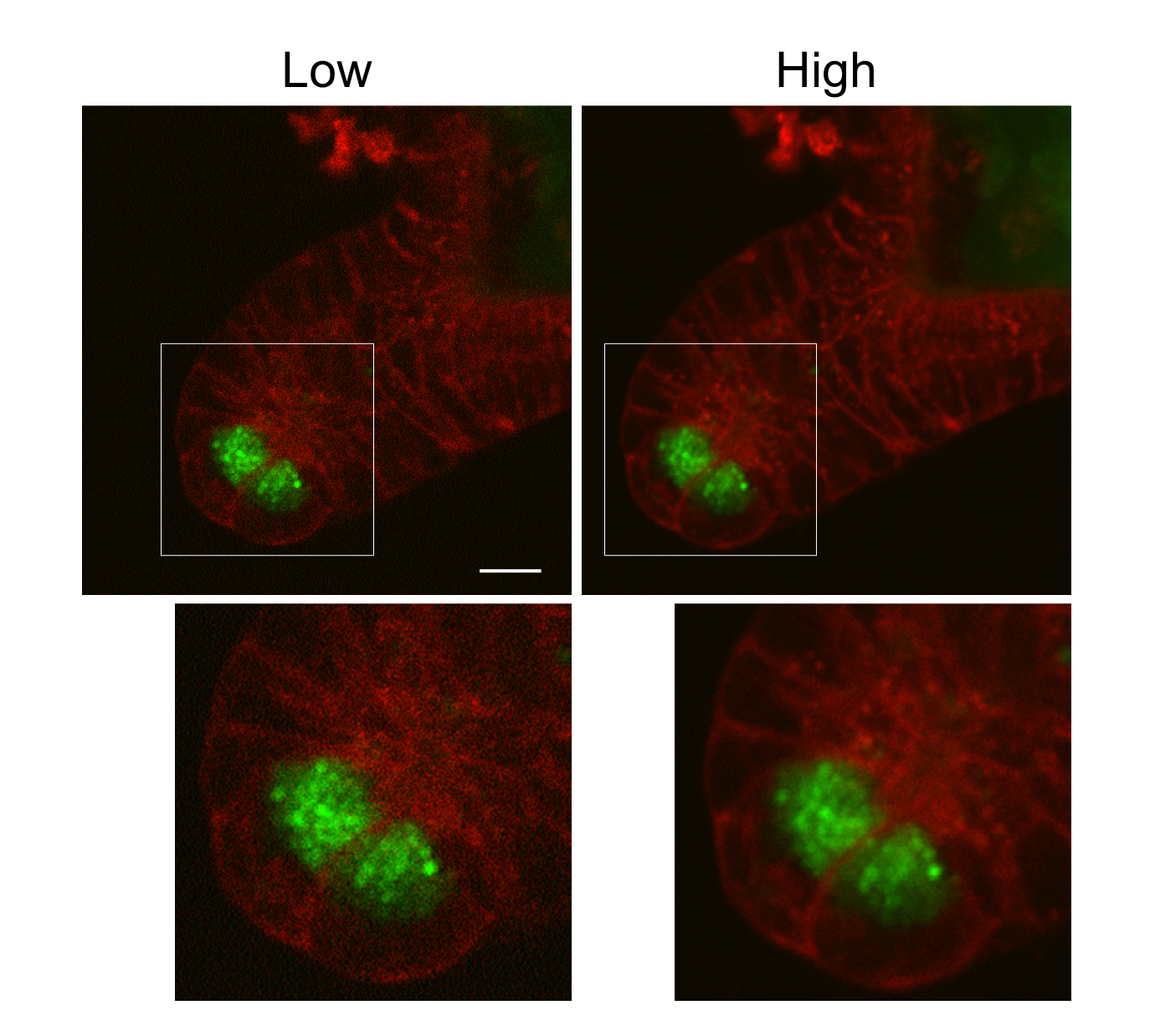

Supplement: S1 Fig — Representative images of enteroids acquired by low-dose light (intermittent-scan) and high-dose light (continuous-scan) illumination conditions. Enteroids were stained by Zinpyr-1 (Paneth cell granule, green) and CellMask (cell membrane, red). Scale bars: 10 μm. (TIF) [file pone.0313213.s001.tif]

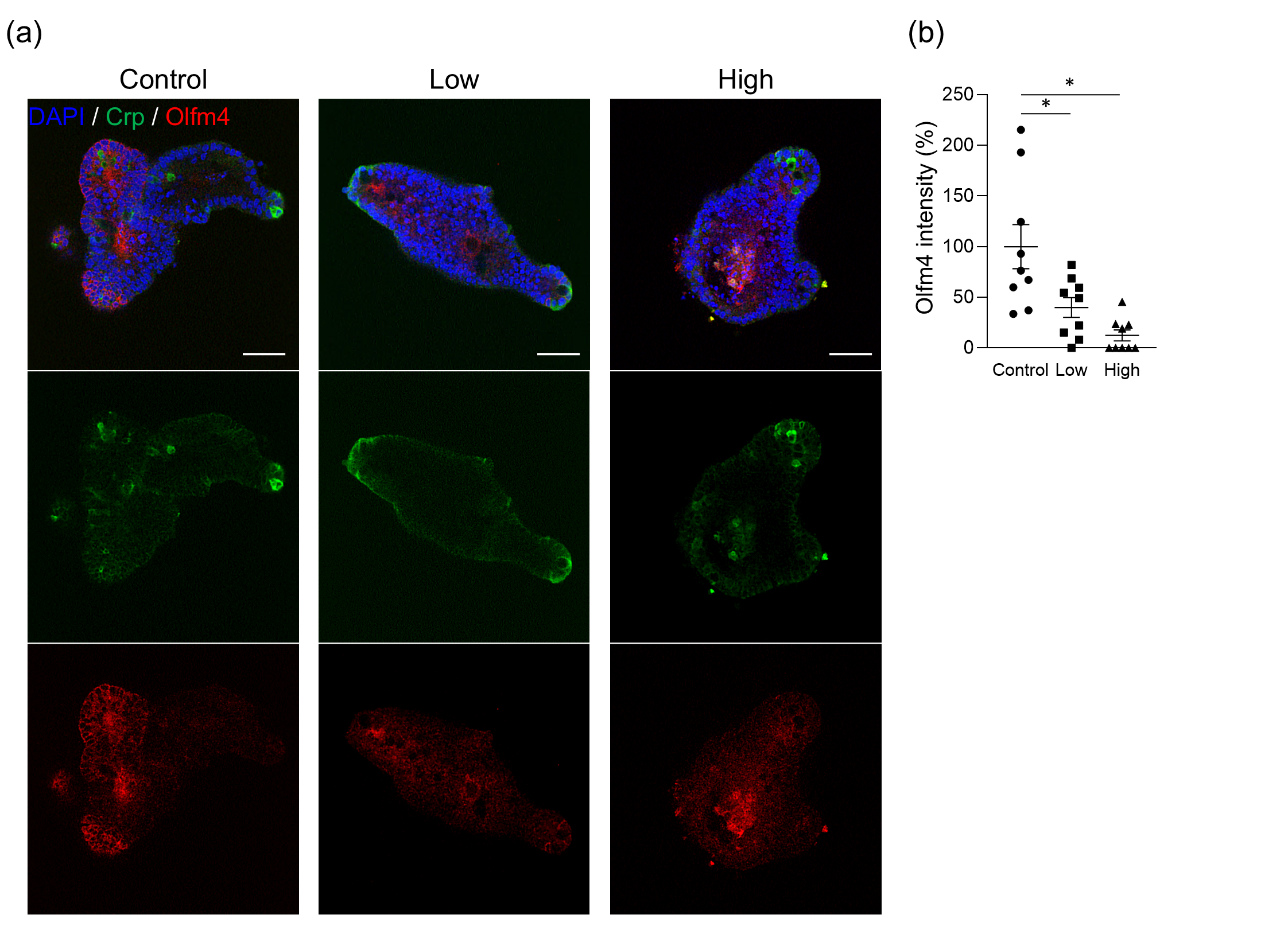

Supplement: S2 Fig — (a) Representative immunofluorescent staining images of Olfm4 (aISC, red) and α-defensin (Paneth cell, green) with DAPI (nucleus, blue) counterstaining in enteroids with each light-illuminated condition. Scale bars: 50 μm. (b) The Olfm4 intensities of light-illuminated enteroids. The values were depicted as mean ± standard error of the mean for three independent experiments. *P < 0.05. (TIF) [file pone.0313213.s002.tif]

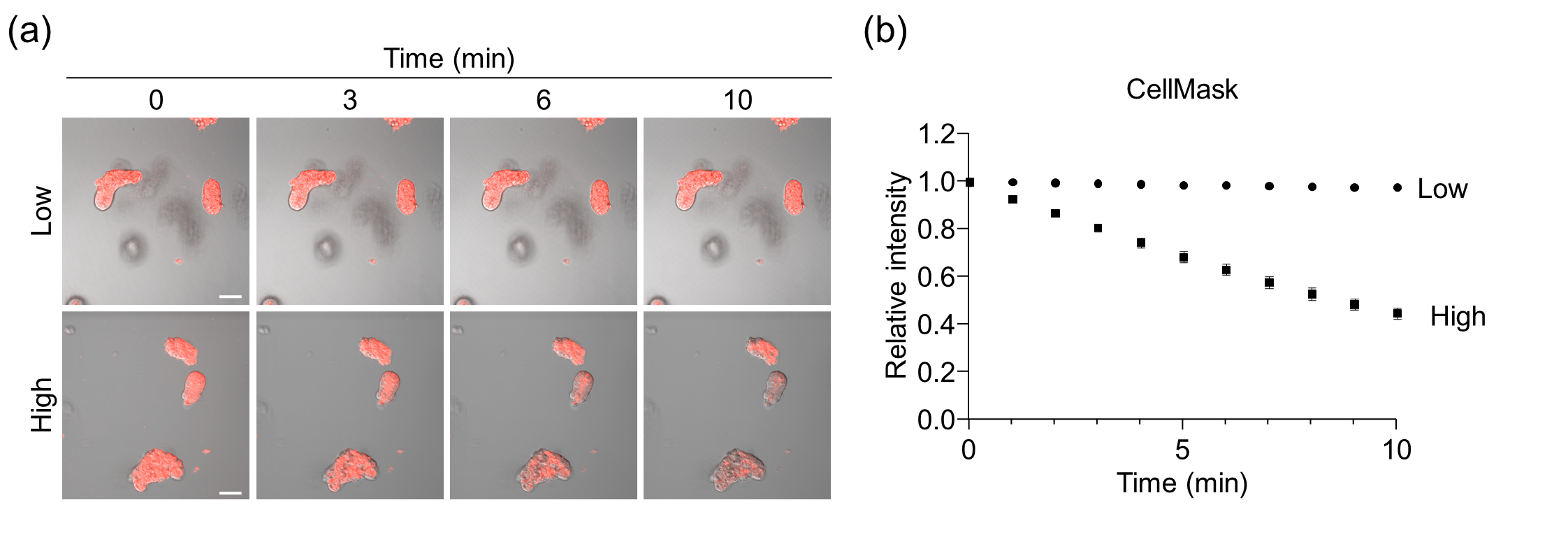

Supplement: S3 Fig — (a) Representative time-lapse images of isolated crypts stained with CellMask exposed to intermittent- or continuous-scan. Scale bars: 50 μm. (b) The time course of CellMask intensity changes in light exposed isolated crypts relative to time = 0 min. The values were depicted as mean ± standard error of the mean. (TIF) [file pone.0313213.s003.tif]
